# Persuasive Messages for Improving Adherence to COVID-19 Prevention Behaviors: Randomized Online Experiment

**DOI:** 10.2196/41328

**Published:** 2023-02-13

**Authors:** Mehdi Mourali, Jamie L Benham, Raynell Lang, Madison M Fullerton, Jean-Christophe Boucher, Kirsten Cornelson, Robert J Oxoby, Cora Constantinescu, Theresa Tang, Deborah A Marshall, Jia Hu

**Affiliations:** 1 Haskayne School of Business University of Calgary Calgary, AB Canada; 2 Department of Medicine Cumming School of Medicine University of Calgary Calgary, AB Canada; 3 Department of Community Health Sciences Cumming School of Medicine University of Calgary Calgary, AB Canada; 4 Department of Political Science School of Public Policy University of Calgary Calgary, AB Canada; 5 Department of Economics University of Notre Dame Notre Dame, IN United States; 6 Department of Economics Faculty of Arts University of Calgary Calgary, AB Canada; 7 Department of Pediatrics Cumming School of Medicine University of Calgary Calgary, AB Canada

**Keywords:** COVID-19, messaging, persuasion, behavioral intentions, public health, compliance, prevention, physical distance, mask, sick, effectiveness, behavior

## Abstract

**Background:**

Adherence to nonpharmaceutical interventions for COVID-19, including physical distancing, masking, staying home while sick, and avoiding crowded indoor spaces, remains critical for limiting the spread of COVID-19.

**Objective:**

The aim of this study was to test the effectiveness of using various persuasive appeals (deontological moral frame, empathy, identifiable victim, goal proximity, and reciprocity) at improving intentions to adhere to prevention behaviors.

**Methods:**

A randomized online experiment using a representative sample of adult Canadian residents with respect to age, ethnicity, and province of residence was performed from March 3 to March 6, 2021. Participants indicated their intentions to follow public health guidelines, saw one of six flyers featuring a persuasive appeal or no appeal, and then rated their intentions a second time. Known correlates of attitudes toward public health measures were also measured.

**Results:**

Intentions to adhere to public health measures increased in all appeal conditions. The message featuring an empathy appeal resulted in a greater increase in intentions than the control (no appeal) message. Moreover, the effectiveness of persuasive appeals was moderated by baseline intentions. Deontological, empathy, identifiable victim, and reciprocity appeals improved intentions more than the control message, but only for people with lower baseline intentions to adhere to nonpharmaceutical interventions.

**Conclusions:**

Public health marketing campaigns aiming to increase adherence to COVID-19 protective behaviors could achieve modest gains by employing a range of persuasive appeals. However, to maximize impact, it is important that these campaigns be targeted to the right individuals.

**Trial Registration:**

ClinicalTrials.gov NCT05722106; https://clinicaltrials.gov/ct2/show/NCT05722106

## Introduction

### Background

As of July 2022, over 500 million people worldwide have contracted the SARS-CoV-2 virus, resulting in over 6 million COVID-19–related deaths [1]. Despite the remarkable and ongoing effort to inoculate the world population (over 12 billion vaccine doses have been administered so far), the rapidly evolving virus continues to spread at alarmingly high rates. Even affluent countries like Canada—a G7 member with over 83% of the population fully vaccinated—are struggling to contain the spread, with case and hospitalization numbers reaching all-time highs in the winter of 2022 [2,3]. With governments gradually lifting restrictive measures and reopening borders, it is critical that, in addition to getting vaccinated, individuals continue to follow nonpharmaceutical interventions—including wearing face masks, physical distancing, staying home when ill, and avoiding crowded indoor spaces—to limit the spread of this highly transmissible virus, especially as newer more transmissible variants continue to emerge [4-7].

Mandates and government-imposed restrictions are important policy tools for limiting the spread of COVID-19, but they are insufficient on their own and must be complemented by softer interventions designed to increase compliance with public health guidelines. Convincing citizens to freely adhere to social distancing, masking, and other preventive behaviors requires persuasive communication going beyond providing information on the risks of the pandemic. Public health organizations and governments need to understand how to best frame messages to effectively appeal to different audiences [8].

The primary objective of this study was to empirically test the effectiveness of message framings emphasizing a set of carefully selected persuasive appeals at improving people’s intentions to engage in health protective behaviors. Another aim of the study was to characterize the target audience most susceptible to respond positively to the persuasive appeals. The findings are intended to guide the design and development of public health campaigns in Canada.

### Message Framing and Adherence to Public Health Measures

In the past year, numerous studies have investigated the impact of various persuasive appeals on people’s attitudes and intentions around COVID-19–related behaviors. The studies varied in their methods and procedures and produced mixed results. Messages using prosocial, altruistic, other-focused, or community-focused appeals were generally more persuasive than messages using self-interested, self-protective, or threatening appeals [9-16]. Likewise, gain-framed messages were typically more effective than loss-framed messages [17,18], although at least one study found the opposite result [19]. Moreover, messages invoking social norms do not seem to be particularly effective [20,21].

In a comprehensive analysis, Pink and colleagues [21] tested 56 short messages using a wide range of framings, including some of the appeals mentioned above. They found no consistent effects for any of the tested messages. Nevertheless, a message using a reciprocity appeal performed the best in three of their five studies.

The present research adds to this body of work by testing the effectiveness of five appeals (deontological moral frame, empathy, goal proximity, identifiable victim, and reciprocity) at improving people’s intentions to adhere to public health measures. This study differs from prior work in at least two important aspects. First, the pandemic context at the time of our study (early March 2021) is unlike that characterizing the early stages of the pandemic when most previous studies were conducted. At the time of our study, there had been over 880,000 confirmed COVID-19 cases in Canada, including over 22,000 deaths. Vaccine supply was limited with just over 2 million doses administered by March 3, 2021 [2]. Although the daily COVID-19 activity had been declining from mid-January through mid-February, it has leveled off since. The 7-day average was under 3000 new cases a day nationwide, but variants of concern (B.1.1.7 and B1.351) had emerged [22]. Masking in public places was mandated in most jurisdictions, and the public was advised to limit travel and minimize contact with people outside of their household [22]. The difference in context alone may result in notable differences in how people process and respond to various persuasive messages.

Previous experiments have largely neglected the role of baseline attitudes and intentions when testing for differences between messages. In contrast, we expected baseline intentions to have a significant impact on how people respond to persuasive messages. People who are highly compliant to begin with have little room left for improvement. Thus, we expected the effect of persuasive appeals to be stronger among those with relatively lower baseline intentions. This is significant because those who are less compliant with public health measures are a critical target for behavior change.

### Five Persuasive Appeals

This study focused on the impact of five persuasive appeals: deontological moral frame, empathy, identifiable victim, goal proximity, and reciprocity. Deontological moral frames are frequently encountered in the current public discourse; they appeal to the sense of duty and responsibilities we have to our families and communities [23]. Prior research suggests that agents making deontological judgments are perceived to be more trustworthy than agents making utilitarian judgments [24,25], even when they are not actually more trustworthy [26]. Moreover, research using machine learning found that moral identity is a strong predictor of adherence to public health measures [27]. Thus, we expect persuasive appeals that use deontological moral frames to help increase adherence to public health measures.

Empathy—understanding and feeling concerned for vulnerable others—has been found to increase altruism and caring, and to motivate helping behavior [28-30]. Thus, inducing empathy by highlighting that the sick, elderly, and immunocompromised need our help is expected to increase adoption of health protective behaviors [13,15].

Goal-proximity appeals emphasize that better days are approaching. This is important because people’s motivation to comply with public health advice has declined since the pandemic’s early days. A Gallup study tracking social distancing behaviors found that the percentage of Americans practicing social distancing dropped steadily over time, from 75% in April 2020 to 38% in March 2021 [31]. A drop in motivation over the course of goal pursuit is not uncommon when pursuing goals with no clear end states or when the tasks required to achieve the goal are difficult [32]. Fortunately, motivational strength tends to increase as the distance to the goal decreases. The goal-gradient hypothesis holds that people apply more effort and persistence as they get closer to a goal’s end state [33-37]. The third message tested in this study relies on this motivational property.

The fourth message relies on the persuasive power of identifiable victims. The identifiable victim effect refers to people’s propensity to offer more help to specific, identifiable victims rather than to anonymous, statistical victims [38-40]. This effect has been attributed to the fact that identifiable victims evoke more powerful emotional responses than statistical victims [38,41]. The identifiable victim effect also arises because people believe their contribution will have a greater impact on an identified victim than on a large group of unidentified victims [39].

Our fifth message relies on the principle of reciprocity. According to Cialdini [42], “all societies subscribe to a norm that obligates individuals to repay in kind what they have received” (page 76). The reciprocity code is not limited to gifts and favors but also includes concessions, whereby people are more likely to make concessions to those who have made concessions to them [43,44]. Accordingly, our reciprocity message emphasizes the sacrifices health care workers are making to help and protect us, and asks that we return the favor by adhering to health protective behaviors.

### Individual Differences in Compliance With Public Health Measures

We expect persuasive communication to have a greater impact among individuals who have lower initial intentions to adhere with public health measures. This is because individuals who have high initial intentions have little room left for improvement; that is, they are already persuaded and further exposure to persuasive communication is unlikely to change their intentions. From a campaign planning perspective, it is important to identify who these individuals might be so that the messages can be efficiently targeted.

The existing literature points to significant variability in the levels of adherence to public health measures [45-52]. A recent review of 29 empirical studies concluded that greater adherence to public health measures is reliably associated with being older, identifying as female, trusting governments, perceiving COVID-19 as a threat, and accessing information through traditional news media [50]. Variability in uptake of public health behaviors was also linked to differences in political ideology [51,52] and perceived responsibility for others [53]. In this study, we measured these characteristics and examined their associations with baseline intentions.

## Methods

### Participants and Procedure

A representative sample of adult Canadian residents with respect to age, ethnicity, and province of residence was recruited by the research firm Critical Mass between March 3 and March 6, 2021. A description of the study was posted on Lucid Marketplace, a third-party platform that maintains an online research panel of 15 million verified users. Users from Canada were invited to visit a screening page assessing demographic and geographic variables. Target quotas for province of residence, age, gender, and ethnicity were set to obtain a demographically representative sample based on the 2016 census data (see Table S1 in Multimedia Appendix 1 for details on the quota system).

Upon consenting in writing, participants reported on their intentions to engage in a set of prevention behaviors over the coming weeks (T1). They were then randomly assigned to an active control or one of five persuasive appeal conditions (control vs deontological vs empathy vs goal proximity vs reciprocity vs identifiable victim) and reported on their intentions to engage in the same set of prevention behaviors a second time (T2). This design allowed us to examine whether the effectiveness of persuasive appeals varies as a function of initial prevention intentions. Finally, participants completed a series of questions assessing potential correlates of prevention intentions. These included measures of political orientation, trust in institutions, perceived threat of COVID-19, and perceived responsibility toward others.

### Ethics Approval

This study was approved by the University of Calgary Conjoint Research Ethics Board (REB21-0173) and was conducted according to the principles expressed in the Declaration of Helsinki.

### Measures

Index variables for intentions to engage in prevention behaviors (pre- and posttreatment) were created by averaging across six items: (1) Limit my physical contact with others when possible, (2) Completely avoid any unnecessary physical contact with others (eg, hugging or handshakes), (3) Avoid crowded indoor spaces, (4) Wear a mask when I leave the house, (5) Wash my hands as much as possible, and (6) Stay home when mildly sick. These items were measured on 100-point sliding scales (0=*strongly disagree*, 50=*neither agree nor disagree*, 100=*strongly agree*).

**Figure 1:**
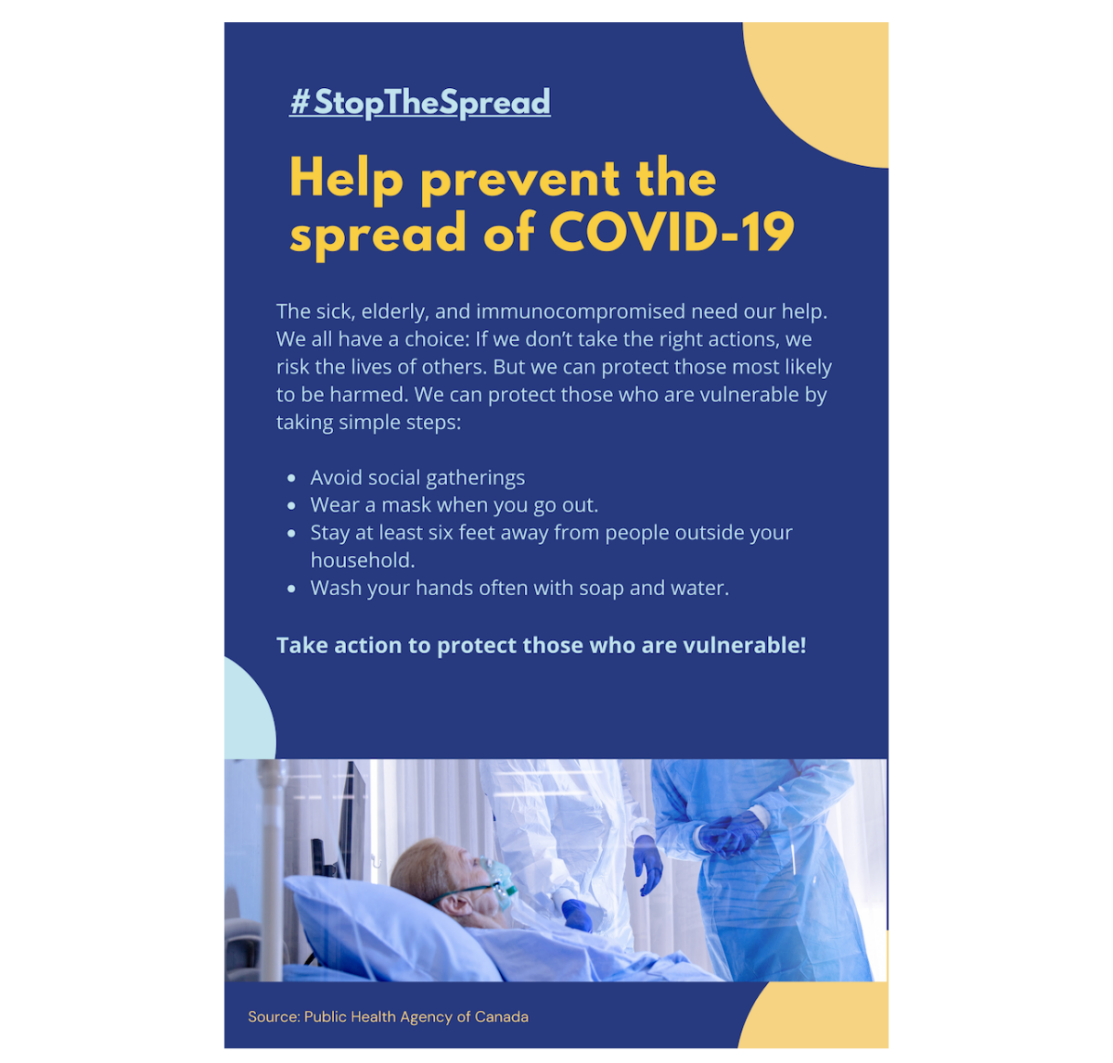
Sample flyer: empathy appeal.

Persuasive appeals were manipulated using promotional flyers ostensibly distributed by the Public Health Agency of Canada. In the control condition, the flyer contained a simple list of what participants can do to help prevent the spread of COVID-19. In each of the five persuasion conditions, the flyer contained the same basic information and a unique persuasive appeal (see Figure 1 for an example and Figures S1-S5 in Multimedia Appendix 1 for the remaining flyers). The wording of the messages is shown in Textbox 1.

Trust in various institutions (politicians, civil servants, public health officials, physicians, other health care providers [eg, nurses, pharmacists], scientists, journalists, and pharmaceutical companies) was measured using eight items (α=.91) on 100-point sliding scales (0=*do not trust at all*, 100=*trust completely*).

Perceived COVID-19 threat was measured using four items (α=.89) adapted from previous research [11]. A sample item is: “To what extent are you afraid of contracting COVID-19 because of the consequences for you personally/your community?” (0=*not at all*, 50=*to a moderate extent*, 100=*to an enormous extent*).

Perceived responsibility toward others was assessed using four items (α=.94) adapted from previous research [18]. A sample item is: “I owe it to my family to do whatever I can to stop the spread of COVID-19” (1=*strongly disagree*, 7=*strongly agree*).

Finally, political orientation was measured using the following item: “If you think about your own political views, where would you classify your views on this scale?” (1=*very liberal*, 7=*very conservative*).

**Messages across appeal conditions.**

**Control**

The virus spreads mainly between people who are in close contact with one another. You can help prevent the spread of COVID-19. We can all do our part:

- Avoid social gatherings.
- Wear a mask when you go out.
- Stay at least six feet away from people outside your household.
- Wash your hands often with soap and water.

These actions prevent the spread of COVID-19.

**Deontological**

The virus spreads mainly between people who are in close contact with one another. You can help prevent the spread of COVID-19. We can all do our part:

- Avoid social gatherings.
- Wear a mask when you go out.
- Stay at least six feet away from people outside your household.
- Wash your hands often with soap and water.

We all need to do this, however difficult, because it is the right thing to do: it is our duty and responsibility to protect our families, friends, and fellow citizens.

**Empathy**

The sick, elderly, and immunocompromised need our help. We all have a choice. If we don’t take the right actions, we risk the lives of others. But we can protect those most likely to be harmed. We can protect those who are vulnerable by taking simple steps:

- Avoid social gatherings.
- Wear a mask when you go out.
- Stay at least six feet away from people outside your household.
- Wash your hands often with soap and water.

Take action to protect those who are vulnerable!

**Identifiable victim**

A few weeks ago, Sam was a healthy 26-year-old with no medical complications. Then he suddenly came down with a bad cough and a feeling like he could not breathe. He tested positive for COVID-19 and is now hospitalized, receiving oxygen from a ventilator, and fighting for his life. This could be any of us. Reduce the risk to yourself and others:

- Avoid social gatherings.
- Wear a mask when you go out.
- Stay at least six feet away from people outside your household.
- Wash your hands often with soap and water.

If we take these actions, we can prevent more people from suffering the way Sam has.

**Goal proximity**

The recent development of safe and effective vaccines gives us great hope. We see the light at the end of the tunnel, but we are not quite there yet. Until a large proportion of the population is immunized, we must remain vigilant and double our efforts to prevent the spread of COVID-19.

- Avoid social gatherings.
- Wear a mask when you go out.
- Stay at least six feet away from people outside your household.
- Wash your hands often with soap and water.

These actions prevent the spread of COVID-19.

**Reciprocity**

Doctors, nurses, and other health care workers are working around the clock, often risking their lives to care for patients with the coronavirus. Working long hours in highly infectious environments, many of them are falling ill. As our health care workers put their lives on the line, we can do our part:

- Avoid social gatherings.
- Wear a mask when you go out.
- Stay at least six feet away from people outside your household.
- Wash your hands often with soap and water.

Our brave health care workers have sacrificed to help others. We should take action too.

### Data Analysis

First, we sought to address the broad question: does exposure to messages using persuasive appeals improve intentions to engage in prevention behaviors more than exposure to the control message? Given the structure in our data (each participant provided two sets of ratings), we fitted a linear mixed effects model (estimated using maximum likelihood) with intention to engage in prevention behaviors as the outcome variable; random intercepts for participants (id); and fixed effects for appeal condition, time of rating, and their interaction. In this analysis, the *P* values were estimated via *t*-tests using the Satterthwaite approximation to degrees of freedom. Effect sizes for the fixed effects are indicated by the standardized regression coefficients (β) and their 95% CIs.

We performed a series of moderated regressions (estimated using ordinary least squares [OLS]) to investigate whether the effectiveness of persuasive appeals varies as a function of baseline prevention intentions. We used change in intentions as the outcome variable, persuasion appeal as a binary predictor, and baseline intentions as a continuous moderator.

To help characterize the target audience, we examined the association of baseline intentions with demographic variables, including age, gender, ethnic background, education, and geographic region, as well as attitudinal variables such as perceived COVID-19 threat, perceived responsibility toward others, trust in institutions, and political orientation.

We fitted a linear model (estimated using OLS) using all predictors. The continuous predictors (age, threat, responsibility, trust, and political orientation) were mean-centered and the categorical predictors were dummy-coded. The ethnic background variable was constructed by recoding the original ethnicity variable into a binary variable (0=ethnic majority, 1=ethnic minority). Education was modified by combining the “less than high school” and “high school” categories into a single “high school or less” category, which served as the baseline group in the analysis. The region variable was constructed by collapsing the Newfoundland and Labrador, Nova Scotia, New Brunswick, and Territories categories in the province variable into a single “Maritimes and Territories” category. Ontario was set as the baseline category for the five-level region variable and female was set as the baseline category for the three-level gender variable.

Data analysis was performed using the statistical program R version 4.0.2 [54], and the level of statistical significance was set at α=.05.

## Results

### Participant Characteristics

A total of 7079 respondents visited the screening page. Of those, 3746 qualified for the main study based on the quota requirements. Of the qualified respondents, 78 failed to complete the survey, resulting in a final sample of 3668 participants (see Table 1 for sample characteristics). Those who failed to complete the survey were demographically similar to those who completed the survey, but were predominantly from the provinces of Quebec (40%) and Nova Scotia (19%) (see Table S1 in Multimedia Appendix 1).

**Table 1:** Sample characteristics.

Characteristic			Overall (N=3668), n (%)		Control (n=582), n (%)		Deontological (n= 622), n (%)		Empathy (n=624), n (%)		Proximity (n= 603), n (%)		Reciprocity (n=623), n (%)		Victim (n=614), n (%)		*P* value^a^	
**Gender (n=3668)**																		.27
	Female	2202 (60.03)		334 (57.4)		380 (61.1)		353 (56.6)		359 (59.5)		386 (62.0)		390 (63.5)				
	Male	1450 (39.53)		245 (42.1)		238 (38.3)		267 (42.8)		243 (40.3)		234 (37.6)		223 (36.3)				
	Other	16 (0.44)		3 (0.5)		4 (0.6)		4 (0.6)		1 (0.2)		3 (0.5)		1 (0.2)				
**Age group (years) (n=3667)**																		.73
	18-24	345 (9.41)		54 (9.3)		65 (10.5)		60 (9.6)		52 (8.6)		54 (8.7)		60 (9.8)				
	25-34	690 (18.82)		118 (20.3)		118 (19.0)		125 (20.0)		115 (19.1)		113 (18.1)		101 (16.4)				
	35-44	785 (21.41)		119 (20.4)		125 (20.1)		146 (23.4)		145 (24.0)		131 (21.0)		119 (19.4)				
	45-54	599 (16.33)		97 (16.7)		106 (17.0)		101 (16.2)		86 (14.3)		103 (16.5)		106 (17.3)				
	55-64	595 (16.23)		100 (17.2)		103 (16.6)		91 (14.6)		102 (16.9)		100 (16.1)		99 (16.1)				
	65-99	653 (17.81)		94 (16.2)		105 (16.9)		101 (16.2)		103 (17.1)		121 (19.4)		129 (21.0)				
**Ethnicity (n=3650)**																		.28
	White	2840 (77.81)		448 (77.0)		479 (77.0)		478 (76.6)		465 (77.1)		488 (78.3)		482 (78.5)				
	Black	110 (3.01)		21 (3.6)		21 (3.4)		18 (2.9)		16 (2.7)		15 (2.4)		19 (3.1)				
	East Asian	297 (8.14)		48 (8.3)		49 (7.9)		63 (10.1)		44 (7.3)		47 (7.6)		46 (7.6)				
	South Asian	193 (5.29)		29 (5.0)		27 (4.4)		34 (5.5)		30 (5.0)		42 (6.8)		31 (5.1)				
	Indigenous	63 (1.73)		12 (2.1)		13 (2.1)		10 (1.6)		6 (1.0)		11 (1.8)		11 (1.8)				
	Other	147 (4.03)		21 (3.6)		29 (4.7)		18 (2.9)		40 (6.7)		19 (3.1)		20 (3.3)				
**Education (n=3667)**																		.23
	Less than high school	86 (2.35)		12 (2.1)		11 (1.8)		12 (1.9)		14 (2.3)		26 (4.2)		11 (1.8)				
	High school	718 (19.58)		112 (19.2)		108 (17.4)		127 (20%)		107 (17.7)		121 (19.4)		143 (23.3)				
	Some college	631 (17.21)		98 (16.8)		111 (17.8)		101 (16%)		113 (18.7)		100 (16.1)		108 (17.6)				
	College	834 (22.74)		128 (22.0)		155 (24.9)		125 (20.4)		143 (23.7)		149 (23.9)		134 (21.8)				
	University	1007 (27.46)		169 (29.0)		174 (28.0)		185 (29.6)		156 (25.9)		165 (26.5)		158 (25.7)				
	Graduate degree	391 (10.66)		63 (10.8)		63 (10.1)		74 (11.9)		69 (11.4)		62 (10.0)		60 (9.8)				
**Province (n=3668)**																		.79
	Newfoundland and Labrador	74 (2.02)		6 (1.0)		18 (2.9)		13 (2.1)		9 (1.5)		14 (2.2)		14 (2.3)				
	Prince Edward Island	19 (0.52)		3 (0.5)		5 (0.8)		4 (0.6)		4 (0.7)		2 (0.3)		1 (0.2)				
	New Brunswick	96 (2.62)		11 (1.9)		20 (3.2)		21 (3.4)		15 (2.5)		10 (1.6)		19 (3.1)				
	Nova Scotia	122 (3.33)		22 (3.8)		23 (3.7)		24 (3.8)		16 (2.7)		22 (3.5)		15 (2.4)				
	Quebec	472 (12.87)		72 (12.4)		87 (14.0)		83 (13.3)		76 (12.6)		66 (10.6)		88 (14)				
	Ontario	1555 (42.39)		256 (44.0)		259 (41.6)		251 (40.2)		267 (44.3)		273 (43.8)		249 (40.6)				
	Manitoba	155 (4.23)		26 (4.5)		26 (4.2)		26 (4.2)		26 (4.3)		30 (4.8)		21 (3.4)				
	Saskatchewan	128 (3.49)		18 (3.1)		21 (3.4)		22 (3.5)		18 (3.0)		20 (3.2)		29 (4.7)				
	Alberta	470 (12.81)		76 (13.1)		77 (12.4)		84 (13.5)		65 (10.8)		86 (13.8)		82 (13.4)				
	British Columbia	569 (15.51)		89 (15.3)		85 (13.7)		95 (15.2)		105 (17.4)		100 (16.1)		95 (15.5)				
	Territories^b^	8 (0.22)		3 (0.5)		1 (0.2)		1 (0.2)		2 (0.3)		0 (0)		1 (0.2)				

^a^Pearson *χ*^2^ test.

^b^Territories=Yukon, Northwest Territories, and Nunavut.

### Intentions to Engage in Prevention Behaviors

The results of the fixed factors in the mixed effects model are summarized in Table 2 (random effects: σ^2^=18.90, τ_00id_=282.54, intraclass correlation coefficient=0.94, N_id_=3668, observations=7331, marginal *R^2^*=0.006, conditional *R^2^*= 0.938). Prior to exposure to the persuasive appeals, participants in all conditions reported similarly high intentions to engage in prevention behaviors. Prevention scores at T1 did not differ significantly between any appeal condition and the control condition, as shown in Table 2 (*P* values for deontological, empathy, goal proximity, reciprocity, and victim are all greater than .05). This confirmed that random assignment produced groups with equivalent baselines. Furthermore, exposure to a reminder message about prevention behaviors (ie, control condition) increased participants’ intentions to engage in prevention behaviors (see Time [T2] variable in Table 2). Additionally, exposure to a persuasive message using an empathy appeal resulted in a larger increase in intentions to engage in prevention behaviors relative to the control message (Table 2).

**Figure 2:**
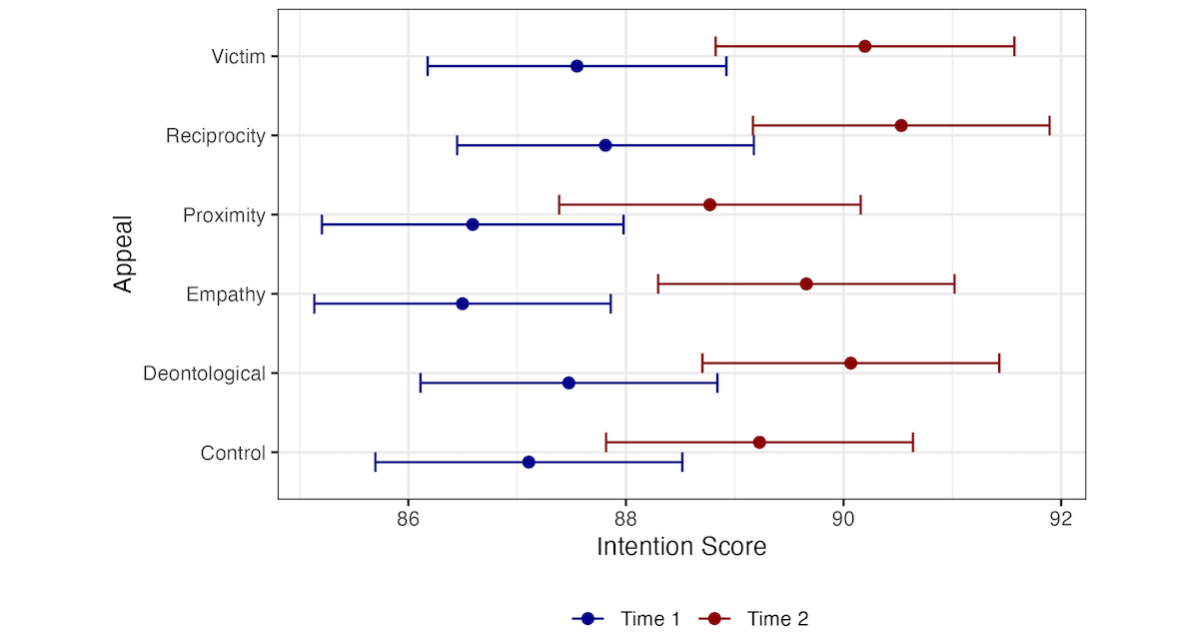
Intention to engage in prevention behaviors across appeal conditions and measurement. Data are presented as marginal means with 95% CIs.

Exposure to messages using other types of appeals (deontological, goal proximity, reciprocity, and victim) produced positive changes in intentions to engage in prevention behaviors (see Table 3), but these changes did not differ in magnitude from those produced by exposure to a simple reminder message (all *P*>.05). Figure 2 shows the estimated marginal means for each group and their 95% CIs.

**Table 2:** Mixed effects regression results for intentions to engage in prevention behaviors.

Predictors	Estimate, b (SE)	*t* statistic	*df*	*P* value	β (95% CI)
(Intercept)	87.11 (0.72)	121.04	3905.10	<.001	–.08 (–.16 to .00)
Time [T2^a^]	2.12 (0.25)	8.32	3663.20	<.001	.12 (.09 to .15)
Deontological	0.37 (1.00)	0.37	3905.10	.71	.02 (–.09 to .13)
Empathy	–0.61 (1.00)	–0.61	3905.10	.54	–.03 (–.15 to .08)
Proximity	–0.52 (1.01)	–0.51	3905.10	.61	–.03 (–.14 to .08)
Reciprocity	0.71 (1.00)	0.70	3905.10	.48	.04 (–.07 to .15)
Victim	0.44 (1.00)	0.44	3905.10	.66	.03 (–.09 to .14)
T2×Deontological	0.47 (0.35)	1.33	3663.38	.19	.03 (–.01 to .07)
T2×Empathy	1.04 (0.35)	2.93	3663.38	.003	.06 (.02 to .10)
T2×Proximity	0.06 (0.36)	0.17	3663.57	.87	.00 (–.04 to .04)
T2×Reciprocity	0.60 (0.35)	1.69	3663.38	.09	.03 (–.01 to .07)
T2×Victim	0.53 (0.36)	1.48	3663.20	.14	.03 (–.01 to .07)

^a^T2: posttest time point.

**Table 3:** Intention to engage in prevention behaviors before (T1) and after (T2) exposure to various appeals.

Appeal	Intention_T1	Intention_T2	T2–T1	*t* statistic	*df*	*P* value
Control	87.1	89.2	2.1	8.83	581	<.001
Deontological	87.5	90.1	2.6	10.83	620	<.001
Empathy	86.5	89.7	3.2	11.73	622	<.001
Proximity	86.6	88.8	2.2	8.71	600	<.001
Reciprocity	87.8	90.5	2.7	11.86	621	<.001
Victim	87.5	90.2	2.7	10.10	613	<.001

### Moderating Effect of Baseline Intentions

The preceding analysis suggested that, apart from empathy, the use of persuasive appeals does not improve intentions to engage in prevention behaviors beyond a simple reminder message. However, we expected the effectiveness of persuasive appeals to vary according to people’s initial dispositions. Persuasive appeals are likely effective when baseline intentions are relatively low, but may have a limited impact when baseline intentions are so high that there is little room for improvement. Results from the moderated regressions were consistent with our expectations (see Table 4). The appeal×baseline intentions interaction was statistically significant for all but the goal-proximity appeal, suggesting that the effectiveness of the deontological, empathy, reciprocity, and identifiable victim appeals indeed depends on the level of initial intentions.

**Figure 3:**
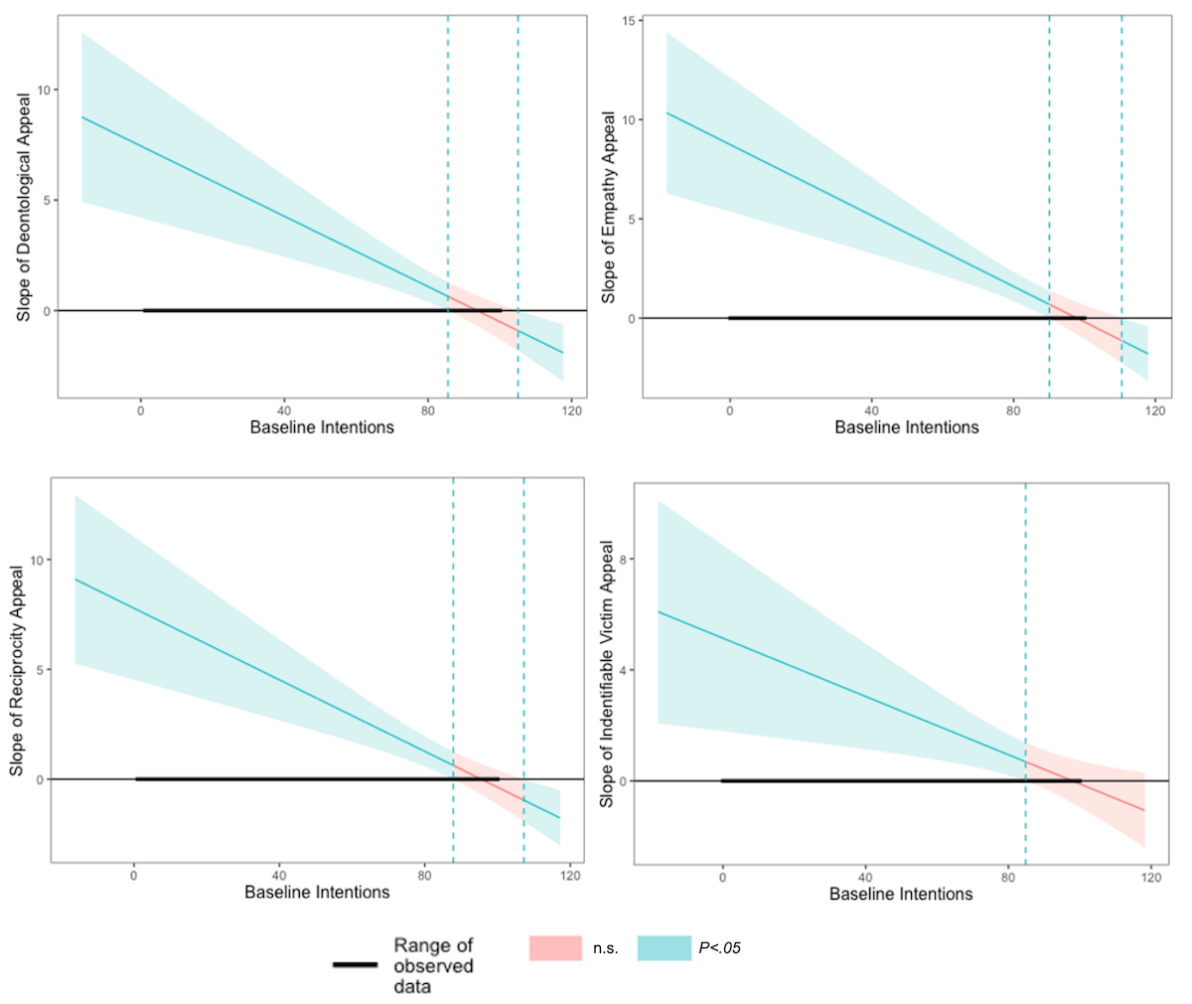
Floodlight analysis of the interactive effects of appeal and baseline intentions. n.s: not significant (*P*>.05).

We followed up with floodlight analyses [55] of each significant interaction. As shown in Figure 3, the conditional effect of seeing a deontological appeal was significant only among participants who had a score of 85.5 or below on the initial intentions measure (30.2% of participants; mean 66.4). In other words, people with lower baseline intentions increased their intentions to engage in prevention behaviors more after seeing a message featuring a deontological appeal than after seeing a message featuring a simple reminder. In contrast, those with high baseline intentions (higher than 85.5; 69.8% of participants; mean 96.2) did not differ significantly in how much they changed their intentions when they saw a message featuring a deontological appeal or a message featuring a reminder.

We observed similar patterns with the other appeals. The conditional effect of empathy was significant only among participants scoring 90.1 or lower on initial intentions (39.5% of participants; mean 71.5), the conditional effect of reciprocity was significant only for those scoring 87.8 or lower on initial intentions (44.1% of participants; mean 68.7), and the conditional effect of identifiable victim was only significant for those scoring 84.8% or lower on initial intentions (29.3% of participants; mean 65.7).

**Table 4:** Effect of appeal×initial intentions interaction on change in intentions to engage in prevention behavior.

Appeal×baseline intentions	Estimate, b (SE)	*t* statistic	*df*	*P* value	β (95% CI)
Deontological	–0.08 (0.02)	–4.29	1199	<.001	–.12 (–.17 to –.06)
Empathy	–0.09 (0.02)	–4.60	1201	<.001	–.13 (–.18 to –.07)
Proximity	–0.02 (0.02)	–1.14	1179	.26	–.03 (–.09 to –.02)
Reciprocity	–0.08 (0.02)	–4.38	1200	<.001	–.12 (–.18 to –.07)
Victim	–0.05 (0.02)	–2.75	1192	.006	–.08 (–.13 to –.02)

### Predictors of Baseline Intentions

The moderation analysis implied that a public health campaign using persuasive appeals would be most effective when targeting individuals with lower baseline intentions: but who might these individuals be?

The regression model using all demographic and attitudinal predictors explained a statistically significant and substantial proportion of the variance (*R^2^*=0.51, *F*_16, 3415_=224.2, *P*<.001, adjusted *R^2^*=0.51). As shown in Table 5, baseline intentions increased with age, perception of COVID-19 threat, perceived responsibility, and trust in institutions. Conversely, baseline intentions decreased with political conservatism, were lower for males relative to females, and were lower in the Prairies compared to Ontario. Neither education level nor ethnic background was significantly uniquely associated with baseline intentions to engage in prevention behaviors.

**Table 5:** Multivariable regression model of initial intentions.

Predictors	Estimate, b (SE)	*t* (*df*=3415)	*P* value	β (95% CI)
(Intercept)	88.44 (0.57)	155.99	<.001	.08 (.01 to .14)
Age	0.06 (0.01)	4.41	<.001	.06 (.03 to .08)
Gender [Male]	–1.74 (0.44)	–3.94	<.001	–.10 (–.15 to –.05)
Gender [Other]	–3.01 (3.19)	–0.95	.34	–.17 (–.53 to .18)
Ethnic [Minority]	–0.07 (0.54)	–0.13	.90	–.00 (–.06 to .06)
Education [Some college]	–0.23 (0.68)	–0.35	.73	–.01 (–.09 to .06)
Education [College]	–0.40 (0.63)	–0.63	.53	–.02 (–.09 to .05)
Education [University]	0.38 (0.61)	0.63	.53	.02 (–.05 to .09)
Education [Graduate degree]	–0.24 (0.79)	–0.30	.76	–.01 (–.10 to .07)
Region [Maritimes]	–1.16 (0.79)	–1.46	.14	–.07 (–.16 to .02)
Region [Quebec]	–0.22 (0.67)	–0.33	.74	–.01 (–.09 to .06)
Region [Prairies]	–1.53 (0.57)	–2.68	.007	–.09 (–.05 to –.02)
Region [British Columbia]	–1.02 (0.63)	–1.64	.10	–.06 (–.13 to .01)
Political orientation	–0.39 (0.14)	–2.76	.006	–.03 (–.06 to –.01)
COVID-19 threat	0.15 (0.01)	13.53	<.001	0.21 (.18 to .24)
Responsibility	7.66 (0.24)	31.90	<.001	.50 (.47 to .53)
Trust	0.08 (0.01)	6.04	<.001	.09 (.06 to .12)

## Discussion

At the time of writing, Canada was entering the fourth wave of COVID-19, with case and hospitalization numbers projected to spike in the coming weeks [2,22]. Maximizing vaccination coverage is paramount, but support for public health measures, including physical distancing, masking, staying home while sick, and avoiding crowded indoor spaces, is also critical for limiting the spread of the virus. This is particularly important since some jurisdictions have moved away from mandatory to recommended measures, relying on the public to make adherence decisions. There is an urgent need for effective messaging to increase adherence to public health measures.

Through a randomized online experiment, we tested the effectiveness of five messages featuring different persuasive appeals (deontological vs empathy vs goal proximity vs reciprocity vs identifiable victim) relative to a control message that simply listed the actions participants could take to help prevent the spread of COVID-19. A pretest-posttest design allowed us to assess and compare the change in intentions after exposure to the various messages. The study produced notable insights. First, baseline intentions across all conditions were relatively high (mean 87.18, SD 17.70 on a 100-point scale). Despite our effort to recruit a demographically representative sample, our pool of respondents may have been skewed toward higher compliance. High baseline intentions could also reflect a degree of social desirability bias in the responses. It is worth noting that similarly high levels of self-reported intentions have been observed in prior research [13,21].

Second, exposure to all messages, including the control message, resulted in a small but statistically significant increase in behavioral intentions. Moreover, the message featuring an empathy appeal increased behavioral intentions to a greater extent than the control message. Given how high intentions were to begin with, a small increase should be considered a significant win.

Third, the impact of persuasive appeals on change in intentions depended on how compliant people were in the first place. For those with lower baseline intentions, messages featuring empathy, deontological, reciprocity, and identifiable victim appeals resulted in greater change than the control message. These results are encouraging, as the intended persuasion targets are precisely those who are less compliant with public health measures.

Finally, the study confirmed much of what prior research had found regarding the correlates of public health compliance. Lower baseline intentions were associated with being male, younger, more politically conservative, residing in the Prairies, perceiving lower levels of COVID-19 threat, accepting less responsibility for the well-being of others, and lacking trust in public institutions [49-53]. These results provide a clear and actionable profile of the audiences that need to be targeted to maximize the efficiency of public health campaigns.

While the findings are reasonably informative, it is important to keep the study’s limitations in mind. For instance, the main outcome consisted of self-reported behavioral intentions. Since a gap often exists between intentions and behavior [56], the observed outcomes may not track perfectly with actual behavior. Moreover, as is the case for all studies of this kind, the results are likely context-dependent. The same appeals may produce vastly different responses in different countries and at different times, depending on cultural values and the COVID-19 situation on the ground. Thus, it is important not to overgeneralize when interpreting the results.

Importantly, the study used a single brief exposure to the messages, offering a conservative test of the messages’ persuasive power. Future research could investigate whether more frequent exposure or a prolonged exposure period would have a stronger impact. Future research could also test the impact of varying the message format (eg, video vs audio vs print), medium (eg, social media vs traditional media), and source. While the Public Health Agency of Canada is generally a trusted source [53], some groups may respond more positively to other sources (eg, trusted religious and community leaders). Although the focus of this study has been squarely on persuasive appeals, public health campaigns would do well to customize not only the content of the message but also its source, format, and media to maximize its impact across different audiences.

## Appendix

Multimedia Appendix 1 Quota system details (Table S1) and flyers for the persuasion conditions (Figures S1-S5).

Multimedia Appendix 2 CONSORT checklist.

## Abbreviations

OLS: ordinary least squares
T1: pretest time point
T2: posttest time point
