# Supplementary material for: Persuasive Messages for Improving Adherence to COVID-19 Prevention Behaviors: Randomized Online Experiment
Source: JMIR Hum Factors. 2023 Feb 13;10:e41328. doi: 10.2196/41328 (PMC9972212; doi:10.2196/41328)
Supplement: Multimedia Appendix 1 [file humanfactors_v10i1e41328_app1.docx]

**Multimedia Appendix 1**

**Table S1. Quotas vs. Final Sample**

|  | **Quota**  **(Based on 2016 Census)** | **Final Sample**  **(N= 3668)** | **Incompletes**  **(N = 78)** |
| --- | --- | --- | --- |
| **Province** |  |  |  |
| NL | 1% | 2.0% | 2.6% |
| PEI | 0.5% | 0.5% | 2.6% |
| NB | 2% | 2.6% | 3.8% |
| NS | 2% | 3.3% | 19% |
| QC | 23% | 13% | 40% |
| ON | 39% | 42% | 3.8% |
| MB | 3% | 4.2% | 3.8% |
| SK | 3% | 3.5% | 9.0% |
| AB | 12% | 13% | 15% |
| BC | 13% | 16% | 0% |
| Territories | 1.5% | 0.2% | 0% |
| **Age Group** |  |  |  |
| 18-24 | 14% | 9.4% | 7.7% |
| 25-34 | 16% | 19% | 24% |
| 35-44 | 16% | 21% | 14% |
| 45-54 | 16% | 16% | 22% |
| 55-64 | 17% | 16% | 14% |
| Over 65 | 21% | 18% | 18% |
| **Gender*** |  |  |  |
| Female | 50% | 60% | 58% |
| Male | 50% | 40% | 42% |
| Other |  | 0.4% |  |
| Ethnicity |  |  |  |
| White | 78% | 78% | 81% |
| Black | 3% | 3.0% | 9.1% |
| East Asian | 5% | 8.1% | 2.6% |
| South Asian | 6% | 5.3% | 2.6% |
| Indigenous | 5% | 1.7% | 1.3% |
| Other | 3% | 4.0% | 3.9% |

*The 2016 census had only two categories for sex (male, female).

**Figure S1. Control Appeal.**

**
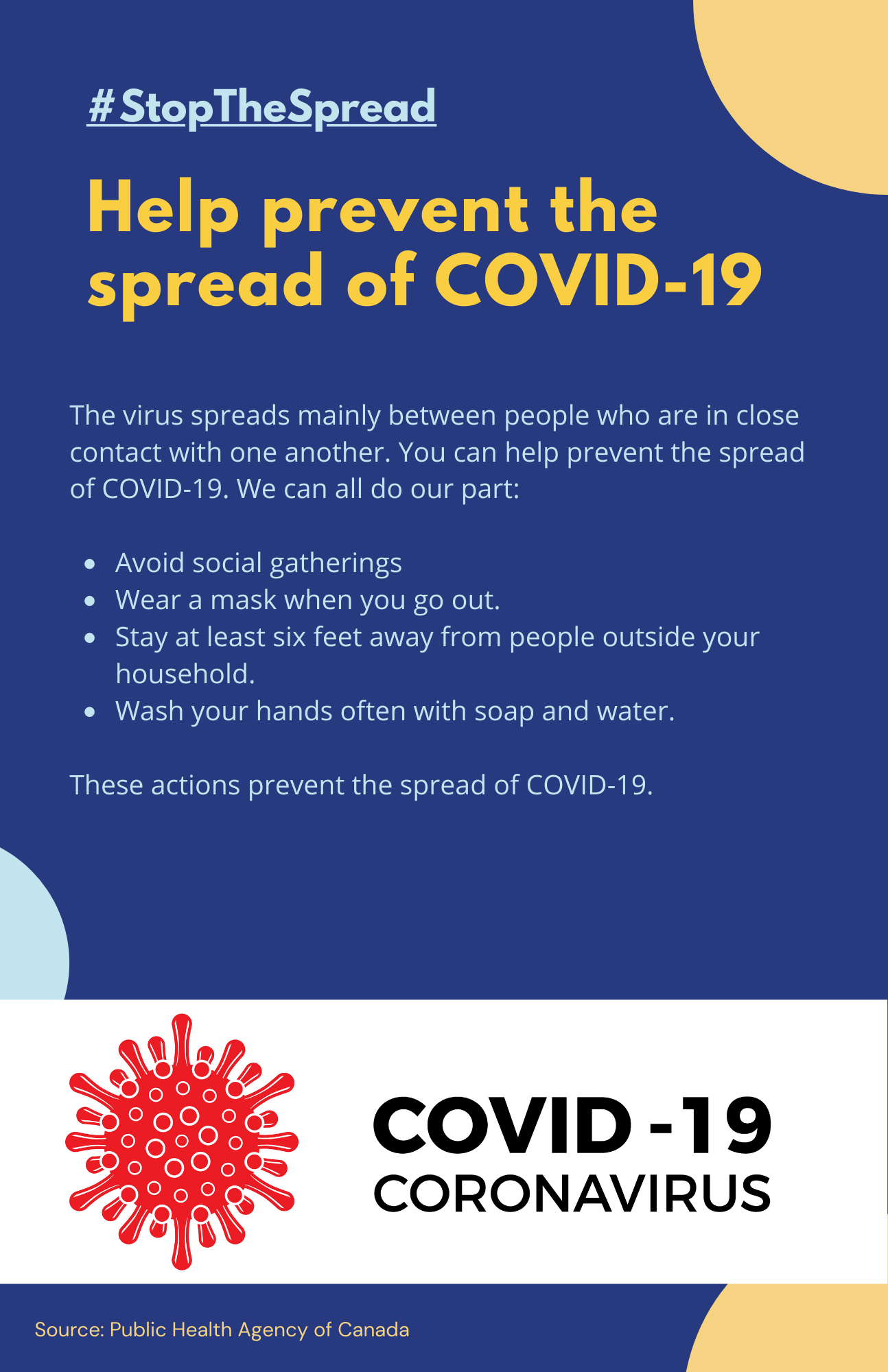
**

**Figure S2. Deontological Appeal**

**
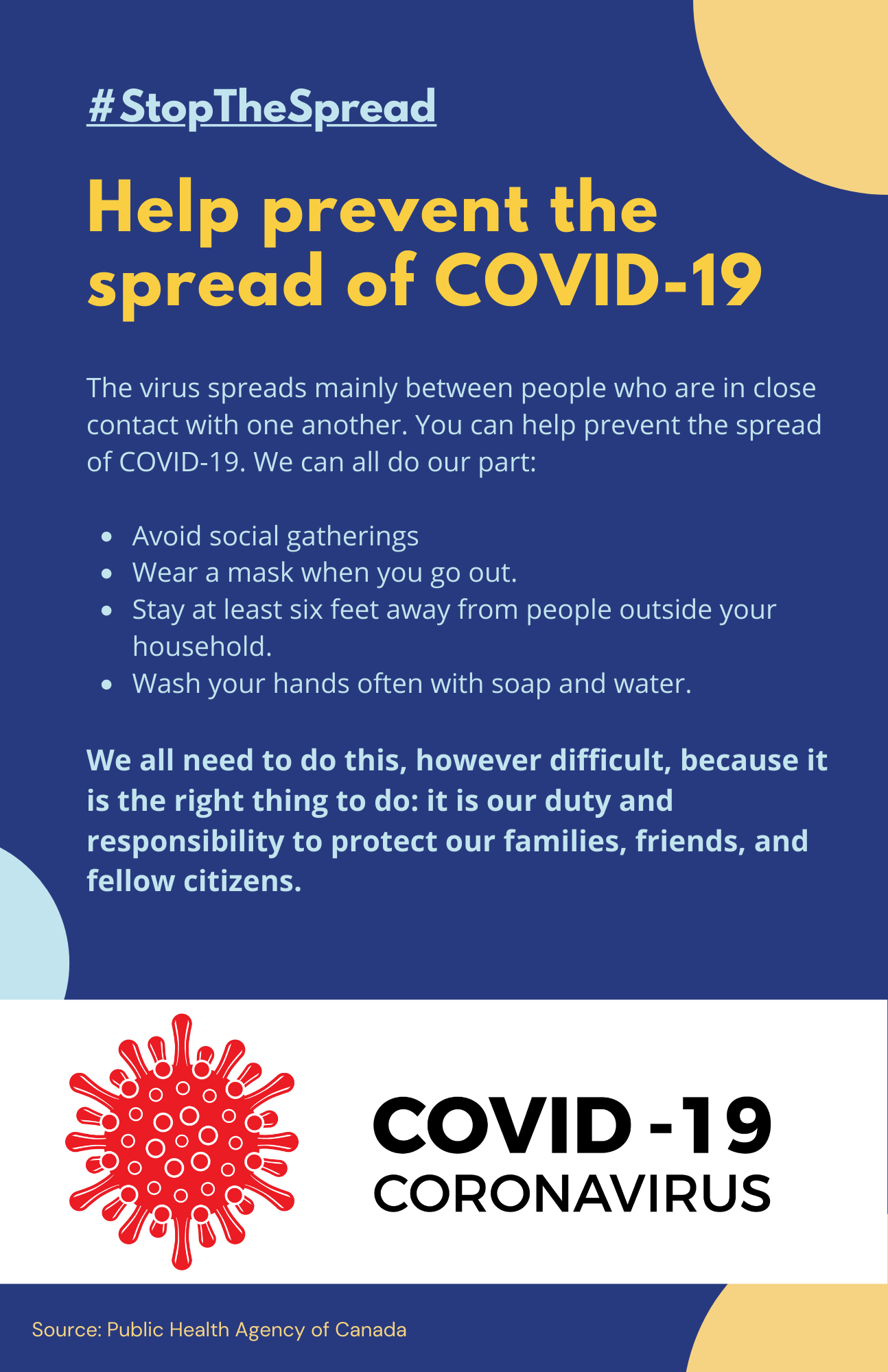
**

**Figure S3. Goal Proximity Appeal**

**
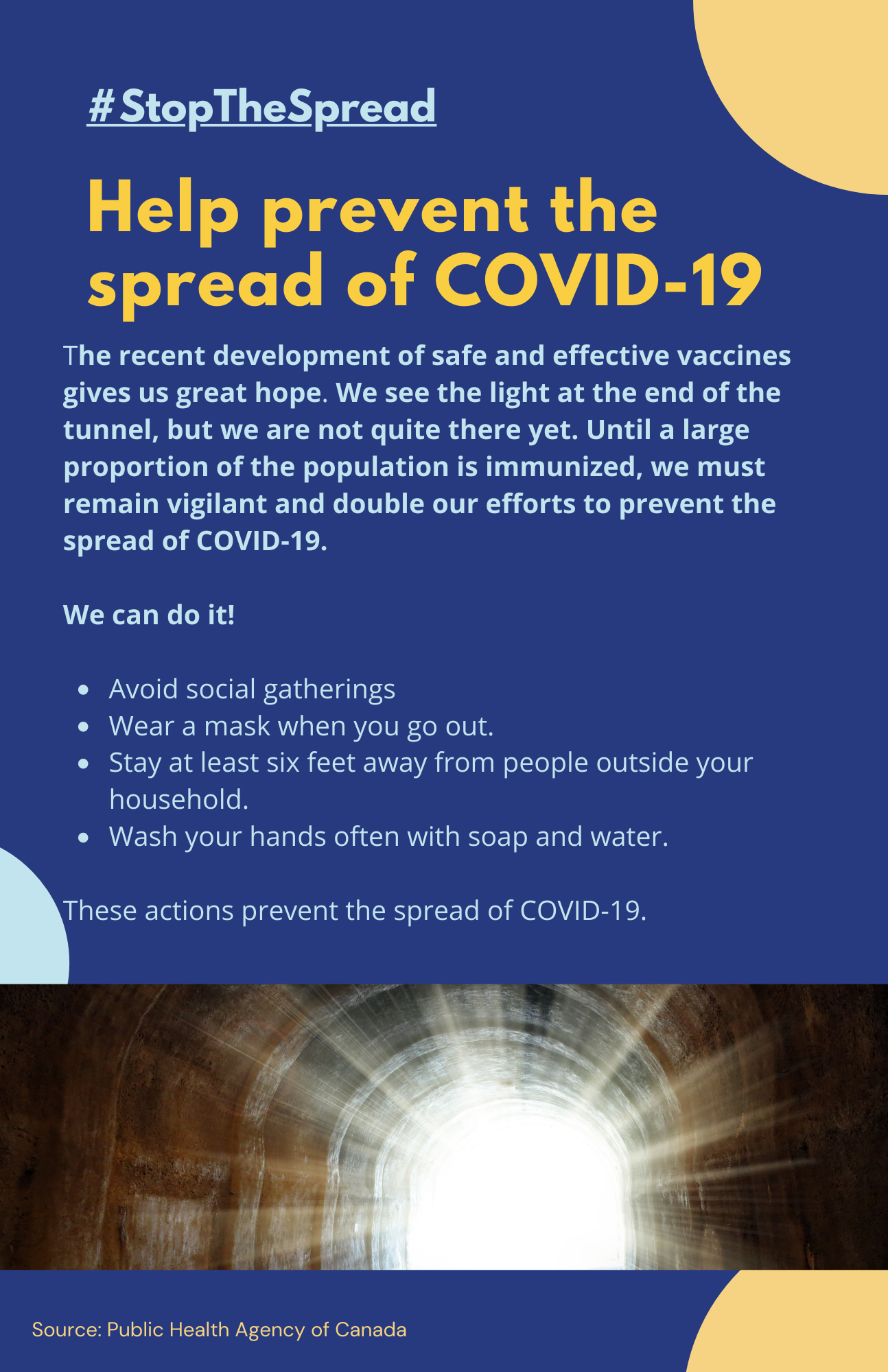
**

**Figure S4. Identifiable Victim Appeal**

**
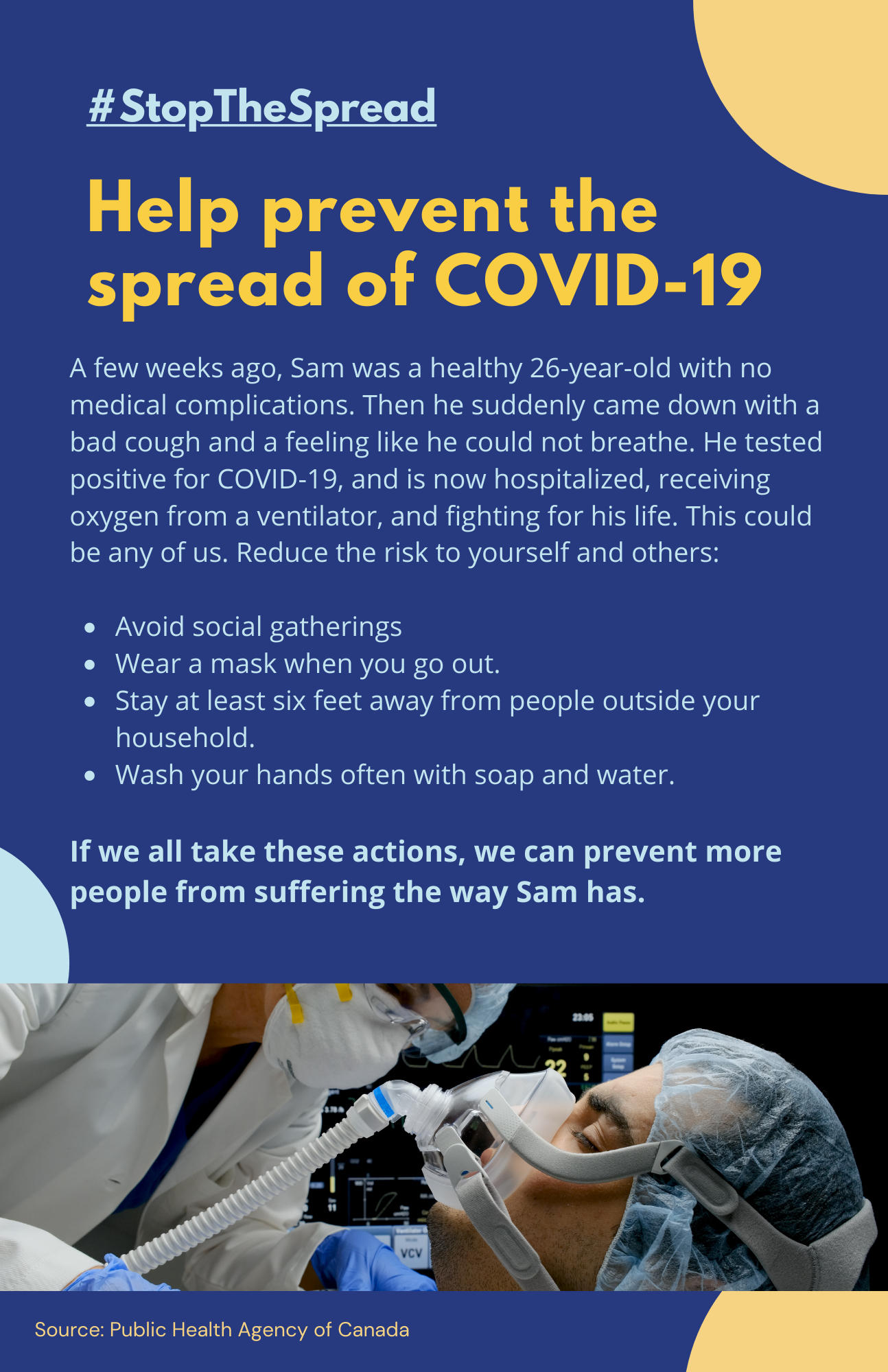
**

**Figure S5. Reciprocity Appeal**

**
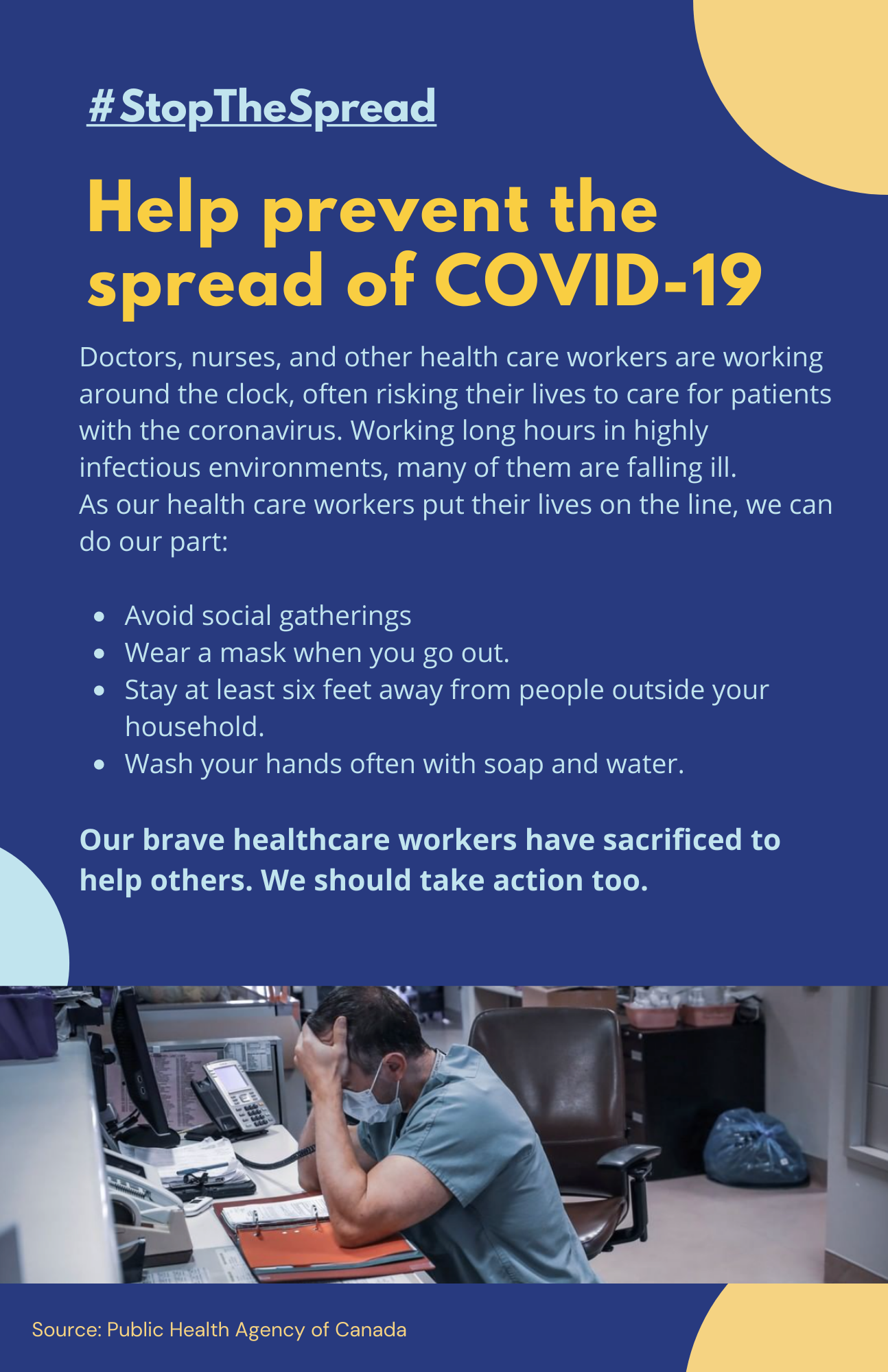
**
